# Supplementary material for: Genome-wide association mapping of bruchid resistance loci in soybean
Source: PLoS One. 2025 Jan 10;20(1):e0292481. doi: 10.1371/journal.pone.0292481 (PMC11723639; doi:10.1371/journal.pone.0292481)
Supplement: S1 Fig — Note, A = percentage weight loss, B = percentage bruchids emergence, C = median development period, and D = Dobie susceptibility index. (DOCX) [file pone.0292481.s006.docx]

(A)





(B)





(C)



(D)

**S1 Fig. QQ plots for GWAS for bruchid resistance traits. Association mapping** **of A = Percentage weight loss, B = Percentage bruchid emergence, C = median development period and, D =Dobie susceptibility index.**
